# Efficacy of Vitreous Biopsy and Clinical Course in Vitreoretinal Lymphoma: A Single-Center Retrospective Analysis

**DOI:** 10.3390/jcm15062344

**Published:** 2026-03-19

**Authors:** Naoya Shiozaki, Tadamichi Akagi, Hiroko Terashima, Takumi Ando, Eriko Ueda, Daigo Kobayashi, Yohei Nozaki, Jun Ominato

**Affiliations:** Division of Ophthalmology and Visual Science, Graduate School of Medical and Dental Sciences, Niigata University, 1-757 Asahimachi-dori, Chuo-ku, Niigata 951-8510, Japan; akagi@med.niigata-u.ac.jp (T.A.); aochan@med.niigata-u.ac.jp (H.T.); takumi.ando@med.niigata-u.ac.jp (T.A.); ipaderi4571@gmail.com (E.U.); m08a034b@med.niigata-u.ac.jp (D.K.); yohe-928-pc@med.niigata-u.ac.jp (Y.N.); am.deluxe.fat.st@gmail.com (J.O.)

**Keywords:** vitreoretinal lymphoma, primary vitreoretinal lymphoma, vitreous biopsy, CNS progression, diagnostic yield

## Abstract

**Background/Objectives**: The high risk of CNS dissemination poses a significant challenge in the management of primary vitreoretinal lymphoma (PVRL). We evaluated the clinical value of our institutional protocol for PVRL, which combines targeted vitreous sampling with routine central nervous system (CNS) surveillance using magnetic resonance imaging (MRI) every 4–6 months. **Methods**: We retrospectively reviewed 34 consecutive patients who underwent vitreous biopsies at Niigata University Hospital between January 2010 and December 2021; 12 patients were initially diagnosed with PVRL without CNS involvement. The protocol mandates submission of both undiluted vitreous samples and the entire vitreous cassette contents, including perfusion fluid, for cytologic evaluation. Patients with PVRL underwent MRI surveillance every 4–6 months. **Results**: Among 12 patients with PVRL, vitreous cytology classified as Class IV or higher demonstrated a positivity rate of 75% (9/12) using undiluted samples alone, which increased to 92% (11/12) when cassette contents were included. Ancillary test results revealed an interleukin (IL)-10/IL-6 ratio > 1 in 75% (9/12) and *immunoglobulin heavy chain* gene rearrangement in 92% (11/12). Extraocular relapse occurred in 92% of patients (11/12), including 10 cases of CNS involvement and one systemic relapse, with a mean time to CNS progression of 11.8 months. The 5-year overall survival was 58%. **Conclusions**: Comprehensive vitreous sampling incorporating perfusion fluid may improve cytologic detection in PVRL within a single-center setting. Routine MRI surveillance facilitates early detection of CNS relapse in patients with PVRL; however, a survival benefit cannot be established from this retrospective analysis.

## 1. Introduction

Vitreoretinal lymphoma (VRL) is a rare malignancy comprising less than 1% of intraocular tumors, and represents a subtype of primary central nervous system lymphoma, most frequently manifesting as diffuse large B-cell lymphoma (DLBCL) [1,2]. A defining and clinically ominous feature is its strong predilection for central nervous system (CNS) involvement, which occurs in approximately 60–90% of patients within several years of diagnosis, resulting in a poor 5-year overall survival of approximately 60% [1,3,4,5]. This high risk of CNS dissemination poses a significant challenge in VRL management.

Clinically, VRL often masquerades as chronic uveitis, presenting with vitreous opacities, subretinal infiltrates, and keratic precipitates, which frequently result in diagnostic delays averaging approximately 21 months and adversely affect prognosis [6,7,8]. Thus, VRL should be strongly considered in the differential diagnosis of atypical or steroid-resistant uveitis to facilitate timely and accurate diagnosis.

Vitreous cytology remains the gold standard for diagnosing VRL; however, its sensitivity is limited to approximately 25.0–44.5% owing to the paucicellular nature of vitreous samples, tumor cell fragility and necrosis, and the confounding effects of prior corticosteroid therapy [9,10]. Therefore, a multimodal approach combining cytology with cytokine analysis (interleukin [IL]-10/IL-6 ratio > 1, sensitivity 89.4%), *immunoglobulin heavy chain* (*IgH*) gene rearrangement analysis, and flow cytometry is indispensable [9,10,11].

The optimal management strategy for primary VRL (PVRL) with no initial CNS involvement remains controversial. Although local therapies, such as intravitreal methotrexate (MTX_ivi_) or radiation, are effective in controlling ocular disease, they do not reliably prevent subsequent CNS relapse [6]. The role of prophylactic systemic chemotherapy, typically high-dose methotrexate regimens, remains debatable, with conflicting results regarding their efficacy [7,8]. A European multicenter retrospective study reported no additional benefit from systemic chemotherapy [12], whereas single-center studies from Japan and the United States have suggested a potential prolongation of time to CNS progression and improvement in progression-free survival [13,14]. These discrepancies may reflect differences in the study design, treatment protocols, and health care systems, particularly the accessibility of frequent neuroimaging modalities, such as magnetic resonance imaging (MRI), in Japan [5].

The current study aimed to retrospectively evaluate the clinical characteristics, diagnostic performance, and treatment outcomes of patients with PVRL managed at our institution, with particular emphasis on the contribution of our comprehensive vitreous sampling protocol to cytologic sensitivity and the role of systematic MRI surveillance in determining the timing of CNS progression compared with previously published reports. Our protocol aimed to improve cytologic detection by maximizing specimen yield at the time of surgery, and the present study evaluates its feasibility in a single-center setting.

## 2. Materials and Methods

### 2.1. Study Design and Patient Cohort

This retrospective observational study was conducted with the approval of the Institutional Review Board (Approval No. 2021-0381) and adhered to the tenets of the Declaration of Helsinki. The requirement for direct informed consent was waived through an opt-out approach, which safeguarded patient privacy while permitting refusal.

We reviewed the medical records of all patients with suspected intraocular lymphoma who underwent vitreous biopsies at the Niigata University Hospital between January 2010 and December 2021. Follow-up data were obtained until December 2024. We excluded patients with secondary intraocular lymphoma (lymphoma with prior extraocular confirmation) or those ultimately diagnosed with non-lymphomatous conditions. Patients without evidence of CNS lesions on initial head MRI were classified as the PVRL group (*n* = 12), whereas those with CNS involvement at or before the time of VRL diagnosis were categorized as the VRL with CNS involvement group (*n* = 8).

### 2.2. Vitreous Sampling Technique

All 25-gauge pars plana vitrectomies were performed according to a standardized protocol designed to maximize specimen yield.

1. Before initiation of infusion, a pre-infusion “dry tap” was performed to aspirate approximately 1 mL of undiluted vitreous, which was allocated for cytokine assay and cytological smear preparation.
2. After initiating infusion, a standard vitrectomy was performed. In cases combined with cataract surgery, separate vitrectomy machines and infusion/aspiration lines were used to prevent contamination of lens-derived material.
3. Following completion of vitrectomy, full cassette contents, which contained resected vitreous and perfusion fluid, were submitted for cytological analysis. Specimens were centrifuged at 2000 rpm (approximately 600× *g*) for 2 min, fixed in Cytorich^®^ Red for 30 min, followed by two washes with purified water, resuspended in 1.0 mL of purified water, and processed for Cytospin slide preparation (800 µL aliquot, 800 rpm), followed by Papanicolaou staining. Since 2010, submission of the entire vitreous cassette contents has been uniformly implemented as part of our institutional diagnostic protocol.

### 2.3. Diagnostic Criteria

A diagnosis of VRL was established if either of the following criteria was fulfilled [8,15]:

1. Positive pathological cytology, defined as Papanicolaou Class IV or V.
2. Fulfillment of at least three of the following four criteria:
  - Pathological cytology classified as Papanicolaou Class III.
  - IL-10/IL-6 ratio > 1 in the vitreous fluid.
  - Positive *IgH* gene rearrangement detected by polymerase chain reaction.
  - Histologically confirmed DLBCL in the CNS or other systemic organs. Cytokine measurements (IL-10 and IL-6) were outsourced to SRL Inc. (Tokyo, Japan). IL-10 was measured using an enzyme-linked immunosorbent assay (ELISA), and IL-6 was measured using a chemiluminescent enzyme immunoassay (CLEIA). Vitreous samples were processed and transported under controlled conditions according to institutional protocols. An IL-10/IL-6 ratio > 1 was considered supportive of VRL based on previously reported literature [16], although this threshold was not prospectively validated in our cohort. Cytokine levels were interpreted in conjunction with cytologic findings and clinical context, recognizing their role as adjunctive rather than standalone diagnostic criteria.

### 2.4. Treatment and Follow-Up Protocols

Local ocular disease was treated with weekly MTX_ivi_ (400 µg/0.1 mL) injections until remission of the vitreous opacities and subretinal infiltrates was achieved. If remission was not achieved after eight weekly injections, the treatment interval was reduced to monthly administration. Local radiation therapy was not selected as a first-line therapy.

Patients with CNS involvement at diagnosis or those who developed CNS progression received systemic chemotherapy, including high-dose methotrexate (HD-MTX) or R-MPV+A (rituximab, methotrexate, procarbazine, vincristine, cytarabine) therapy. Whole-brain radiation therapy was administered at the discretion of the neurosurgeons and radiation oncologists.

Patients in the PVRL group underwent routine asymptomatic CNS surveillance with contrast-enhanced brain MRI (1.5T or 3T) every 4–6 months.

### 2.5. Statistical Analysis

Continuous variables were compared between groups using the Mann–Whitney U test, and categorical variables were compared using Fisher’s exact test. Survival analysis was performed using the Kaplan–Meier method with log-rank testing. Hazard ratios (HRs) were estimated using univariate Cox proportional hazards regression. Although exploratory multivariable Cox regression could theoretically incorporate clinical and molecular variables (e.g., age, laterality, IgH status, IL-10/IL-6 positivity), the limited number of events in our cohort precluded reliable modeling. As a result, only univariate analyses were performed, and the prognostic implications of these variables remain hypothesis-generating.

## 3. Results

**Figure 1:**
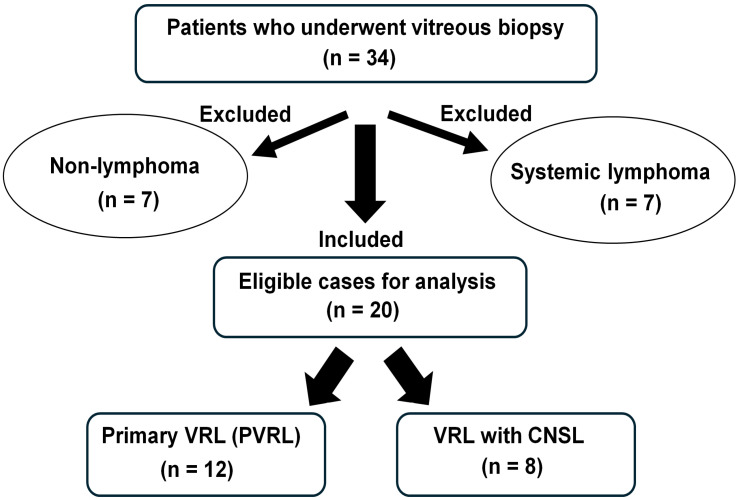
Flowchart depicting the selection of patients who underwent vitreous biopsy for the study. VRL = vitreoretinal lymphoma.

Of the 34 patients who underwent vitreous biopsies, seven with prior extraocular lymphoma and seven with non-lymphoma diagnoses were excluded (Figure 1). Ultimately, 20 patients with VRL were included in this study (Table 1). The mean age was 71.5 years, and 60% (12/20) were female. Bilateral involvement was observed in 70% (14/20) of patients. The PVRL (no initial CNS lesions, *n* = 12) and VRL with CNS involvement (*n* = 8) groups showed no significant differences in age, sex, or laterality.

In the PVRL group, the positivity rate for cytology (Papanicolaou Class IV or V) obtained from the undiluted “dry tap” specimen was 75% (9/12; 95% CI 42.8–94.5%), which increased to 92% (11/12; 95% CI 61.5–99.8%) when the cassette contents were analyzed. The difference was not statistically significant (McNemar exact test, *p* = 0.50). This improvement was clinically significant in two cases: Case 4, in which the dry tap specimen was negative (Class I) but the cassette specimen was positive (Class V), and Case 6, in which the dry tap specimen was suspicious (Class III) but the cassette specimen yielded a definitive diagnosis (Class V) (Table 2). Ancillary diagnostic testing demonstrated *IgH* gene rearrangement positivity in 92% (11/12) and an IL-10/IL-6 ratio > 1 in 75% (9/12) of the patients.

**Figure 2:**
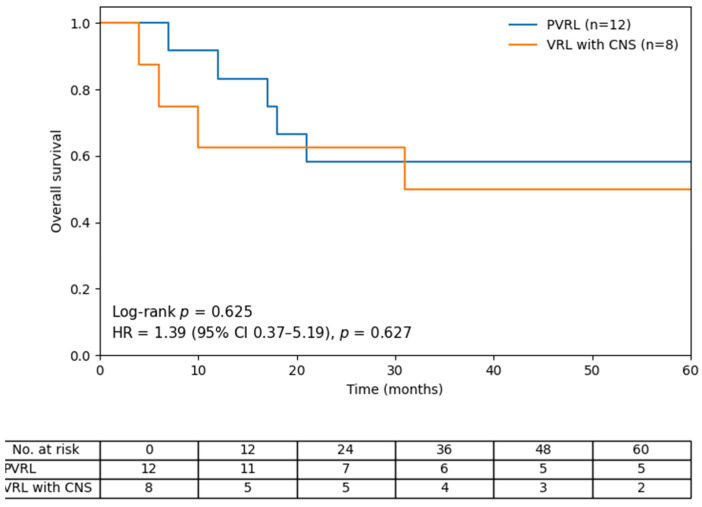
Kaplan–Meier analysis of overall survival in patients with PVRL versus those with VRL and CNS involvement. No. at risk: Number of patients at risk.

The detailed clinical course of each patient is presented in Table 2. In the PVRL group, extraocular progression occurred in 92% (11/12) of the patients, including CNS progression in 10 patients and systemic relapse presenting as malignant pleural effusion in one patient. The mean interval from PVRL diagnosis to extraocular disease progression was 11.8 months (range, 1–45 months). Five patients (42%) died during the observation period. Kaplan–Meier analysis demonstrated a 5-year overall survival rate of 58% in the PVRL group. There was no significant difference in overall survival between the PVRL and VRL with CNS groups (log-rank *p* = 0.625). In univariate Cox regression analysis, concurrent CNS involvement was not significantly associated with mortality (HR 1.39; 95% CI 0.37–5.19; *p* = 0.627) (Figure 2). In the VRL with CNS involvement group, the positivity rate for *IgH* gene rearrangement was lower (50%; 4/8), whereas the positivity rate for an IL-10/IL-6 ratio > 1 was comparable (75%; 6/8) (Table 2).

## 4. Discussion

This single-center study provides two key insights. First, by employing a meticulous vitreous specimen submission protocol that included vitreous perfusion fluid, we achieved a high cytological positivity rate of 92%, even without the use of cell block techniques. Second, the mean time to CNS progression in our cohort was shorter than that reported in previous studies, which we attribute to earlier detection enabled by our systematic MRI screening protocol.

The most notable finding of our study is the relatively high diagnostic yield of 92% for vitreous cytology in patients with PVRL. This rate is higher than the historically reported rates of 25.0–44.5% for conventional smear cytology [17], which has long been limited by the paucicellular nature and fragility of lymphoma cells in vitreous samples. Our success can be attributed to a simple yet critical modification of the sampling protocol: submission of the entire contents of the vitreous cassette in addition to the initial undiluted “dry tap” specimen. The low sensitivity of smear cytology has been attributed to limited cellularity; moreover, larger vitreous volumes have been shown to improve diagnostic yield [18].

An additional technical consideration is the potential impact of concurrent cataract surgery on the cytological results. Previous studies have suggested that combining cataract extraction with vitreous biopsy may increase the likelihood of nondiagnostic specimens due to lens-derived contaminants [19]. To mitigate this risk, we deliberately used separate surgical systems for cataract extraction and vitrectomy. This precaution was intended to ensure that lens fragments or crystallin proteins did not contaminate the vitreous cassette, thereby preserving sample integrity and potentially contributing to the high diagnostic yield observed in our cohort.

The mechanisms underlying vitreoretinal involvement in primary CNS lymphoma (PCNSL) remain incompletely understood. Both the central nervous system and the eye are considered immune-privileged sites, which may permit survival and expansion of malignant B cells within a relatively protected microenvironment. Lymphoma cells are thought to migrate through the retinal vasculature or across the sub-retinal pigment epithelium into the vitreous cavity. Local cytokine production, particularly IL-10, may further contribute to immune modulation and tumor cell survival within the intraocular space. Although our study does not directly investigate pathogenesis, these mechanisms provide biological context for the frequent coexistence and sequential progression between ocular and CNS disease in VRL.

In recent years, the cell block method has emerged as the benchmark for high-sensitivity VRL cytology, substantially improving the diagnostic rates. A landmark study by Kase et al. demonstrated that the cell block technique increased the positivity rate from 35.7% with conventional smears to 93.3% [20]. This finding established the principle that larger volumes of diluted vitreous fluid are superior to small undiluted samples. Our study builds on this concept by demonstrating that a comparable diagnostic yield of 92% can be achieved through a surgeon-driven strategy focused on maximizing cellular yield at the time of surgery, without requiring the more complex laboratory processing. However, it is noteworthy, that this study was not designed as a comparative effectiveness trial, and no direct comparison with conventional smear-only or cell-block techniques was performed. Therefore, superiority or equivalence cannot be concluded from this retrospective single-center analysis.

Our approach aimed not to replace the cell block, but rather to highlight that the absolute number of cells delivered to the pathologist is the most critical determinant of diagnostic success. Both our method and the cell block technique utilize the same source material—large-volume diluted vitreous fluid from the vitrectomy cassette. The fact that our simpler processing achieved a nearly identical success rate suggests that maximizing initial cell harvest during surgery is the important factor influencing diagnostic yield.

This concept is clearly illustrated by individual cases within our cohort. For example, Case 4 yielded a negative result (Class I) on the dry tap but was definitively diagnosed as positive (Class V) using the cassette specimen. Similarly, Case 6 was upgraded from suspicious (Class III) on the dry tap to positive (Class V) via the cassette. These cases represent instances of “diagnostic rescue,” in which submission of the cassette specimen directly prevented missed or delayed diagnoses and obviated the need for repeat invasive biopsy. This finding underscores that meticulous surgical sampling is a critical—and potentially underappreciated—component of the VRL diagnostic pathway. Routine submission of all resected vitreous components may therefore improve diagnostic accuracy and facilitate timely treatment. In our cohort, cytologic findings ranged from Papanicolaou Class I to Class V, with Class IV–V strongly supporting the diagnosis of VRL. Several Class III cases required careful interpretation, and cassette-processed material occasionally provided additional atypical lymphoid aggregates that strengthened diagnostic confidence. Cytokine analysis and IgH rearrangement testing were used as adjunctive tools rather than standalone diagnostic standards. An IL-10/IL-6 ratio >1 was observed in 75% of patients and interpreted as suggestive of lymphomatous activity, particularly in cases with borderline cytology. IgH rearrangement analysis demonstrated monoclonality in 92% of patients, providing critical diagnostic support when cytology was indeterminate. Together, these findings underscore the importance of integrating cytology with cytokine and molecular analyses within a multimodal diagnostic framework.

The mean time to CNS progression in our PVRL cohort was 11.8 months, which is shorter than the 16.2–30 months reported in other studies [21]. This finding should not be interpreted as evidence of more aggressive disease or inferior local treatment efficacy in our cohort. Rather, it likely reflects the effectiveness of our institutional policy of routine head MRI surveillance every 4–6 months, irrespective of neurological symptoms. Although this supports the feasibility of systematic MRI screening for timely identification of CNS relapse, our study does not demonstrate improved survival outcomes, and therefore detection timing must be distinguished from clinical benefit. A recent review emphasized that scheduled MRI surveillance enables early detection of CNS involvement, which ultimately occurs in most patients with PVRL [22]. Consistent with our findings, Kase et al. reported that CNS progression was detected significantly earlier in asymptomatic patients (mean: 6.3 months) than in symptomatic patients (mean 21.1 months) [13]. Our observed mean interval of approximately 12 months likely reflects detection at an early, often asymptomatic, stage.

This observation has important implications for the interpretation of progression-free survival as a study endpoint in VRL. In cohorts undergoing active surveillance, a shorter progression-free survival may not signify a worse prognosis but rather successful early detection. The more critical endpoint is whether early detection followed by timely intervention improves overall survival. Our findings suggest that systematic MRI surveillance enables earlier detection; however, whether this translates into improved survival remains uncertain.

This study has certain limitations.

- First, its retrospective design introduces potential selection bias.
- Second, cytology contributed to the diagnostic criteria, creating possible incorporation bias that may overestimate diagnostic yield. Ancillary tests such as IL-10/IL-6 ratios and IgH rearrangement were not uniformly available and the absence of an independent reference standard further limits diagnostic certainty.
- Third, treatment regimens were heterogeneous. Patients received diverse regimens—including intravitreal chemotherapy, systemic high-dose methotrexate–based regimens, immunochemotherapy, whole-brain radiation, and targeted agents. This variability reflects real-world practice but substantially limits interpretation of survival and progression outcomes. The absence of stratified analysis prevents assessment of regimen-specific efficacy, and therefore our findings should be interpreted as descriptive rather than comparative.
- Fourth, scheduled MRI surveillance may introduce lead-time bias; therefore, earlier detection of CNS progression does not necessarily translate into improved survival.
- Fifth, although cassette-inclusive cytology improved diagnostic yield from 75% to 92%, this represents an absolute increase in only two patients, and the confidence intervals remain wide due to the small sample size. The difference was not statistically significant, and therefore superiority or equivalence to cell-block methods cannot be concluded from this retrospective analysis. These findings should be interpreted as hypothesis-generating and warrant validation in larger, multicenter cohorts.
- Finally, our findings should be interpreted within the context of feasibility rather than comparative effectiveness. The absence of a control group, direct cell-block comparison, or cross-institutional benchmarking limits generalizability. Future multicenter studies with standardized comparative designs are warranted to establish relative performance against established techniques.

## 5. Conclusions

This single-center retrospective study suggests that a meticulous surgical sampling technique, involving the submission of all resected vitreous and perfusion fluid, may achieve a high cytological diagnostic rate of 92% for primary vitreoretinal lymphoma without specialized cell block processing. Furthermore, our results indicate that systematic head MRI surveillance at 4–6-month intervals may facilitate earlier detection of CNS progression. However, given the retrospective design and limited sample size, a definitive survival benefit cannot be established. Standardization of vitreous specimen collection and prospective multicenter studies are warranted to further validate these findings.

## Figures and Tables

**Table 1:** Patient demographics and clinical characteristics.

Feature	PVRL Group (*n* = 12)	VRL with CNS Group (*n* = 8)	Overall (*n* = 20)
Age, years	72.8 ± 8.2 (56–85)	68.6 ± 12.5 (49–83)	71.5 ± 10.0 (49–85)
Sex (Male/Female), No.	3/9	5/3	8/12
Laterality (Bilateral/Unilateral), No.	9/3	5/3	14/6
Length of follow-up, months	36.7 ± 25.1 (7–84)	54.4 ± 45.4 (4–125)	43.8 ± 35.1 (4–125)
Feature	PVRL Group (*n* = 12)	VRL with CNS Group (*n* = 8)	Overall (*n* = 20)

PVRL: primary vitreoretinal lymphoma; VRL with CNS: vitreoretinal lymphoma with central nervous system involvement; SD: standard deviation. *p*-values calculated using Mann–Whitney U test or Fisher’s exact test. Data are mean ± standard deviation (range) unless otherwise indicated.

**Table 2:** Comprehensive Clinical, Pathological, and Prognostic Data for Patients with Vitreoretinal Lymphoma (*n* = 20).

Group	Case No.	Age	Sex	Eye	Decimal VA (R/L)	Cytology (Dry Tap)	Cytology (Cassette)	*IgH* Rearrangement	IL-10/IL-6 > 1	Time to Extraocular Disease (Months)	Site of Progression	Treatment	Status
PVRL (*n* = 12)	1	78	F	B	0.7/0.7	V	V	Positive	Yes	1	CNS	WBRT	Dead
	2	75	F	B	0.8/0.03	V	V	Positive	Yes	20	CNS	HD-MTX	Dead
	3	67	M	B	1.2/1.2	V	V	Negative	Yes	6	CNS	HD-MTX, MPV, WBRT	Alive
	4	85	M	B	0.2/0.5	I	V	Positive	Yes	1	CNS	WBRT	Dead
	5	69	F	B	1.2/1.2	III	III	Positive	Yes	2	CNS	MPV+A, MTX	Alive
	6	74	F	R	0.3	III	V	Positive	N/A	5	CNS	WBRT	Dead
	7	78	F	R	0.8	V	V	Positive	Yes	45	CNS	WBRT	Alive
	8	75	M	B	1.2/0.1	V	V	Positive	No	20	Malignant pleural effusion (systemic relapse)	R-TCOP	Alive
	9	48	F	B	0.8/0.9	V	V	Positive	Yes	5	CNS	MPV+A, WBRT	Alive
	10	82	F	B	1.0/0.01	V	V	Positive	Yes	2	CNS	MTX	Dead
	11	56	F	B	1.2/0.04	V	V	Positive	No	20	CNS	HD-MTX, MPV+A, WBRT, Tir	Alive
	12	58	F	B	1.2/0.6	IV	IV	Positive	Yes	19	CNS	HD-MPV, MPV+A	Alive
VRL with CNS (*n* = 8)	13	75	M	R	1.0/0.7	V	N/A ^b^	Negative	Yes	N/A ^c^	N/A ^c^	HD-MTX, R-CHOP, WBRT	Dead
	14	63	M	B	1.2/0.6	V	V	Positive	Yes	N/A ^c^	N/A ^c^	MPV+A, RT	Dead
	15	83	F	L	0.7	V	V	Negative	N/A	N/A ^c^	N/A ^c^	R-TCOP, RT	Dead
	16	60	M	R	1.2	V	V	Negative	Yes	N/A ^c^	N/A ^c^	MPV, WBRT	Dead
	17	49	M	B	0.6/0.3	V	N/A ^b^	Positive	Yes	N/A ^c^	N/A ^c^	MPV, WBRT	Alive
	18	76	F	R	0.9	V	V	Negative	Yes	N/A ^c^	N/A ^c^	WBRT	Alive
	19	75	F	B	0.2/0.4	V	V	Positive	N/A ^a^	N/A ^c^	N/A ^c^	Subtotal resection, WBRT	Dead
	20	68	M	B	0.5/0.7	V	V	Positive	Yes	N/A ^c^	N/A ^c^	HD-AraC, MPV, WBRT, Tir	Alive

^a^ Cytokine analysis not performed due to insufficient sample volume. ^b^ Cassette specimen was not submitted for analysis in this case. ^c^ Not applicable as patients had CNS involvement at the time of diagnosis. PVRL: primary vitreoretinal lymphoma; VRL with CNS: vitreoretinal lymphoma with central nervous system involvement; *IgH*: immunoglobulin heavy chain; IL: interleukin; CNS: central nervous system; WBRT: whole-brain radiation therapy; HD-MTX: high-dose methotrexate; MPV: methotrexate, procarbazine, vincristine; MPV+A: MPV plus cytarabine; MTX: methotrexate; R-TCOP: rituximab, cyclophosphamide, doxorubicin, vincristine, prednisolone; Tir: tirabrutinib; HD-MPV: high-dose MPV; R-CHOP: rituximab, cyclophosphamide, doxorubicin, vincristine, prednisolone; RT: radiation therapy; HD-AraC: high-dose cytarabine; N/A: not applicable.

## Data Availability

The original contributions presented in this study are included in the article material. Further inquiries can be directed to the corresponding author.

## Abbreviations

The following abbreviations are used in this manuscript:

VRL	Vitreoretinal lymphoma
DLBCL	Diffuse large B-cell lymphoma
CNS	Central nervous system
IL	Interleukin
IgH	Immunoglobulin heavy chain
PVRL	Primary vitreoretinal lymphoma
MTX_ivi_	Intravitreal methotrexate
MRI	Magnetic resonance imaging
HD-MTX	High-dose methotrexate
R-MPV+A	Rituximab, methotrexate, procarbazine, vincristine, cytarabine
